# Antinociceptive and Anti-Inflammatory Activities of Crude Methanolic Extract of Red Alga *Bryothamnion triquetrum*

**DOI:** 10.3390/md10091977

**Published:** 2012-09-17

**Authors:** Luiz Henrique Agra Cavalcante-Silva, Carolina Barbosa Brito da Matta, Morgana Vital de Araújo, José Maria Barbosa-Filho, Daysianne Pereira de Lira, Bárbara Viviana de Oliveira Santos, George Emmanuel C. de Miranda, Magna Suzana Alexandre-Moreira

**Affiliations:** 1 LaFI-Laboratory of Pharmacology and Immunity, Institute of Biological Sciences and Health, Federal University of Alagoas, Maceió 57020-720, AL, Brazil; Email: luiz0710@gmail.com (L.H.A.C.-S.); carolina_damatta@hotmail.com (C.B.B.M.); morgana_vital@hotmail.com (M.V.A.); 2 Laboratory of Technology Pharmaceutical, Federal University of Paraíba, João Pessoa 58051-900, PB, Brazil; Email: jbarbosa@ltf.ufpb.br (J.M.B.-F.); daysianneplira@yahoo.com.br (D.P.L.); 3 Laboratory of Marine Algae, Department of Systematics and Ecology, Federal University of Paraíba, João Pessoa 58051-900, PB, Brazil; Email: mirandag@dse.ufpb.br

**Keywords:** *Bryothamnion triquetrum*, red algae, antinociceptive, anti-inflammatory

## Abstract

The marine environment is an extraordinary reservoir of bioactive natural products, many of which exhibit chemical and structural features not found in terrestrial natural products. In this regard, the aim of this study was to investigate the possible antinociceptive and anti-inflammatory activities of a crude methanolic extract of the red alga *Bryothamnion triquetrum* (BT-MeOH) in murine models. Groups of Swiss mice of both sexes (25–30 g) were used throughout the experiments. The potential antinociceptive of BT-MeOH was evaluated by means of the following tests: acetic acid-induced writhing, hot-plate test and glutamate- and formalin-induced nociception. The anti-inflammatory activity of BT-MeOH was investigated using the zymosan A-induced peritonitis test. The tests were conducted using 100 mg/kg (p.o.) BT-MeOH, 33.3 mg/kg (p.o.) dipyrone, 35.7 mg/kg (p.o.) indomethacin and 5.7 mg/kg (s.c.) morphine. The extract and all standard drugs were administered 40 min before the nociceptive/inflammatory stimulus. In the acetic acid-induced writhing test, BT-MeOH and dipyrone inhibited the nociceptive response by 55.9% (22.2 ± 2.0 writhings; *p* < 0.01) and 80.9% (9.6 ± 2.1 writhings; *p* < 0.01). In the hot-plate test, BT-MeOH did not increase the latency time of the animals in the time evaluated. In addition, BT-MeOH inhibited glutamate-induced nociception by 50.1%. While BT-MeOH did not inhibit the neurogenic phase in formalin-induced nociception, the inflammatory phase was inhibited by 53.1% (66.8 ± 14.2 s; *p* < 0.01). Indomethacin inhibited the inflammatory phase by 60.2% (56.8 ± 8.7 s; *p* < 0.01). In the zymosan-induced peritonitis test, BT-MeOH inhibited 55.6% (6.6 ± 0.2 × 10^6^ leukocytes/mL; *p* < 0.01) of leukocyte migration, while indomethacin inhibited 78.1% (3.2 ± 0.1 × 10^6^ leukocytes/mL; *p* < 0.01). Based on the results obtained in this study, we conclude that BT-MeOH has peripheral antinociceptive and anti-inflammatory activities. However, more studies need to be conducted to confirm these properties.

## 1. Introduction

The oceans are home to 90% of the world’s living biomass, which makes up about half of the total global biodiversity [1]. Thus, the marine environment is an extraordinary reservoir of bioactive natural products, many of which exhibit chemical and structural features not found in terrestrial natural products [2,3,4]. The fact is that marine organisms have evolved physiological and biochemical mechanisms to help them survive in this hostile environment, and because of this, most classes of marine organisms show very structurally different secondary metabolites with unusual structural features [5,6,7]. 

Although the oceans are a rich source of bioactive compounds, they only started to attract interest from pharmaceutical companies and research institutions approximately 50 years ago [8]. Since then, more than 14,000 different natural products from marine organisms have been described, and hundreds of patents describing new bioactive marine natural products have been filed [9,10,11,12]. Several marine natural products are currently in pre-clinical and clinical evaluation, especially in the areas of cancer, pain and inflammatory diseases [13,14,15,16,17].

Among marine organisms, marine macroalgae (more commonly known as seaweeds) are some of nature’s most biologically active resources, as they possess a wealth of bioactive compounds [18,19,20,21,22,23]. Moreover, seaweeds have an advantage over other marine organisms due to their easy availability and great potential for cultivation [24]. However, marine algae have been identified as underutilized plant resources [25]. 

Seaweeds can be classified into three categories, according to their physical structure, pigmentation, function and life cycle: Chlorophyta (green algae), Phaeophyta (brown algae) and Rhodophyta (red algae) [16]. There are about 8000 species of red algae, most of which are of marine sources [26]. They dominate tropical and warm waters, and can also be found in the colder regions of the world [27]. The red color of these algae results from the dominance of the pigments phycoerythrin and phycocyanin, which mask the other pigments, *i.e.*, chlorophyll a, carotenoids and a number of unique xanthophylls [28]. According to some authors, red algae are the most important source of many biologically active metabolites in comparison to other classes of algae [26].

In this regard, recent studies have shown red algae to be a potential source of new lead compounds to treat inflammatory and pain disorders. The main characteristic of these secondary metabolites is the presence of halogens (Cl and Br) [29], such as in neorogioltriol, a tricyclic brominated diterpenoid isolated from the red alga *Laurencia glandulifera*. Neorogioltriol has an analgesic effect by blocking the activation of primary afferents, through a mechanism dependent on the activation of opioid receptors [30], and it has anti-inflammatory properties involving the inhibition of NF-κB transactivation and TNFα release [31]. Other halogen compounds such as vidalols A and B, bromophenols isolated from the red alga *Vidalia obtusaloba*, have a potent anti-inflammatory activity [32]. Furthermore, lectins isolated from red algae have assumed a pivotal role in the discovery of new anti-inflammatory and analgesic drugs [33,34,35].

The mechanisms and mediators involved in painful and inflammatory processes have been the target of several studies in recent years [36,37,38,39]. However, the various natural and synthetic compounds used for the treatment of these processes have not yet been identified as ideal anti-inflammatory or analgesic agents, either due to their limited effectiveness or the magnitude of their adverse effects [40]. In this regard, it is important to note that many anti-inflammatory drugs available today can cause acute gastrointestinal damage, kidney diseases, platelet disorders, hepatotoxicity, pancreatitis and other side effects [41], which represents an important limitation to the use of these drugs. Thus, the search for substances that have potent anti-inflammatory activity and limited adverse effects is still quite encouraged by the scientific community [42,43,44,45,46,47,48,49].

Thus, due to the increasing interest in the study of seaweeds, in addition to the relevance of a study that focuses more broadly on their effect on the nociceptive and inflammatory response induced by different stimuli, the aim of this study was to evaluate the antinociceptive and anti-inflammatory potential of a crude methanolic extract of the red alga *B. triquetrum*, which occurs along the northeast coast of Brazil.

## 2. Results and Discussion

Initially, the acute toxicity of BT-MeOH at a dose of 1000 mg/kg p.o. was evaluated, which was tenfold the therapeutic dose, and no signs of toxicity were observed. This result enabled us to carry out the biological tests safely.

**Figure 1:**
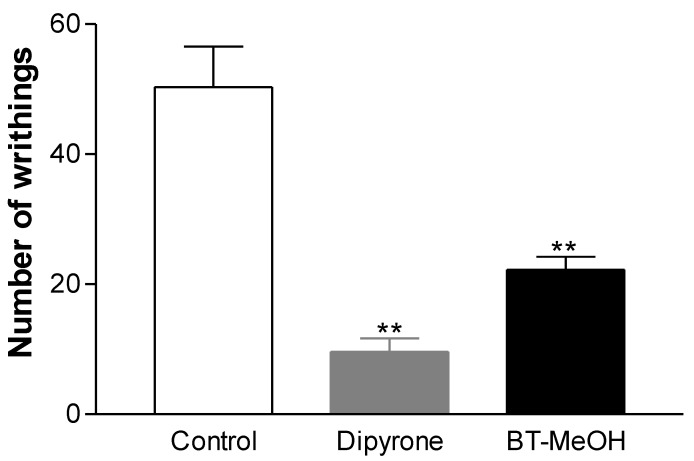
Antinociceptive effect of BT-MeOH (100 mg/kg, p.o.) and dipyrone (33.3 mg/kg, p.o.) in acetic acid-induced writhing. Each column represents the mean ± S.E.M. of six animals. Statistical differences between the treated and control groups were evaluated by ANOVA and Dunnett’s test, and the asterisks denote the level of significance in comparison with control group, ** *p* < 0.01.

To evaluate the ability of extract in modulating nociceptive and inflammatory processes, the first test performed was acetic acid-induced writhing. In this test, the treatment with BT-MeOH, compared with control group (50.3 ± 6.2 writhings), induced inhibition of the nociceptive response by 55.9% (22.2 ± 2.0 writhings; *p* < 0.01). The standard drug dipyrone (33.3 mg/kg, p.o.) inhibited 80.9% (9.6 ± 2.1 writhings; *p* < 0.01) of writhing in mice (Figure 1). 

In this model, after acetic acid injection, the mice show a response characterized by abdominal constriction, which is sometimes accompanied by twisting of the trunk followed by extension of the hind limbs. This behavior results from the activation of ASICs (acid-sensitive ion channels) and TRPV1 localized in afferent primary fibers [50]. Furthermore, acetic acid injection induces a release of TNF-α, interleukin 1β and interleukin 8 by resident peritoneal macrophages and mast cells [51], prostanoids and bradykinin [52,53]. Given that BT-MeOH was active in the writhing test, there were probably metabolites present such as *trans*-cinnamic, *p*-coumaric and ferulic acids that antagonized the nociceptive action of acetic acid.

**Figure 2:**
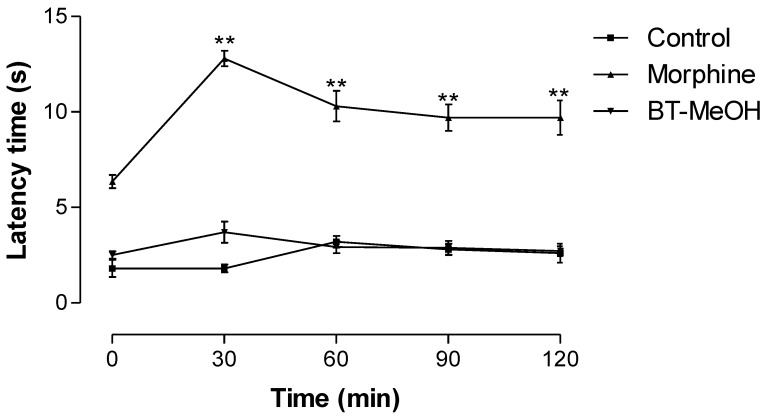
Time course of BT-MeOH (100 mg/kg, p.o.) and morphine (5.7 mg/kg, s.c.) on thermal nociception (hot plate). The results represent the mean ± S.E.M. of six animals. Statistical differences between the treated and control groups were evaluated by ANOVA and Dunnett’s test to assess the significance levels in comparison with zero-time. The asterisks denote the level of significance in comparison with zero-time, ** *p* < 0.01.

Given that the writing test is sensitive to various drugs with a peripheral and central action [54,55,56,57], the hot-plate test was carried out to identify any possible central antinociceptive activity. The treatment with BT-MeOH did not increase the reaction latency time of mice on the hot plate, suggesting that BT-MeOH did not possess central antinociceptive activity. On the other hand, as expected, morphine induced a significant increase in the latency time of mice at all times evaluated (Figure 2). The hot-plate test has been used to evaluate nociceptive activity mediated by central mechanisms. The thermal stimulus induces two kinds of behavior: paw licking and jumping [58,59]. Both result from TRPV activation by heat. Once activated, this ion channel promotes Ca^2+^ influx, which depolarizes sensory fibers and induces voltage-dependent Na^+^ channel opening, triggering an action potential [50].

**Figure 3:**
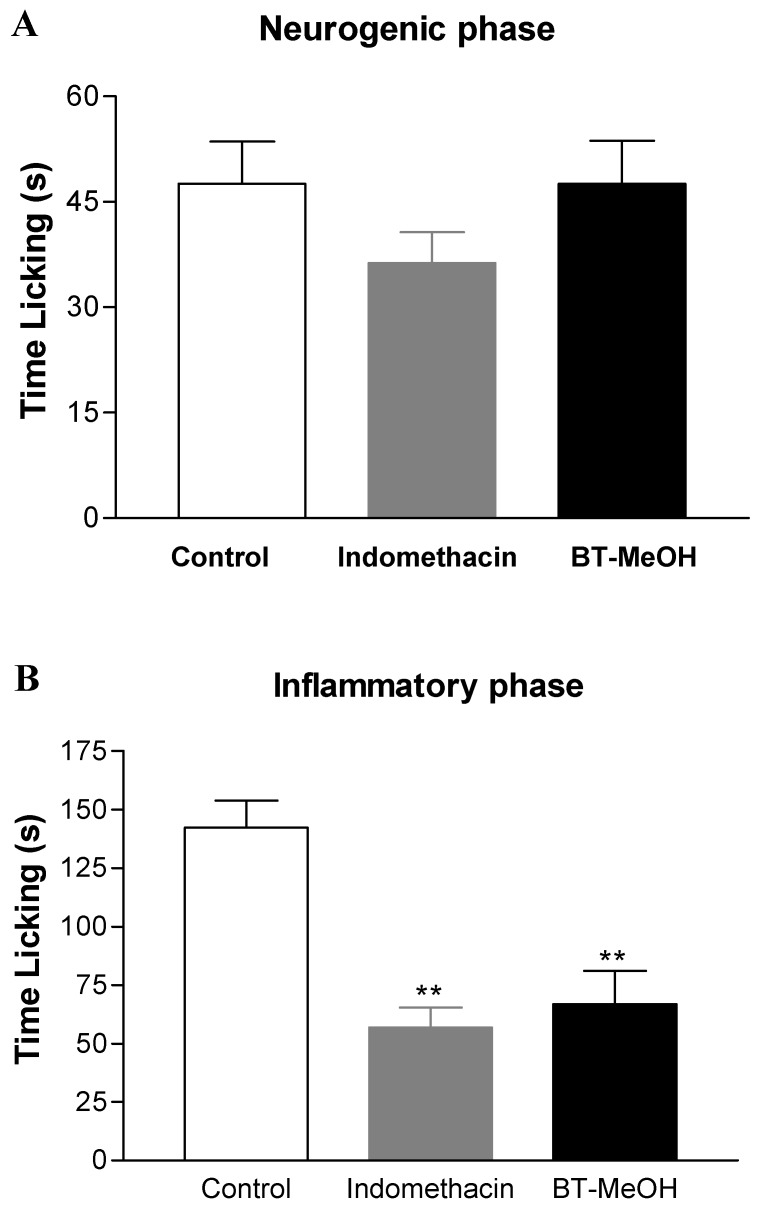
Antinociceptive effect of the BT-MeOH (100 mg/kg, p.o) and indomethacin (35.7 mg/kg, p.o.) on early phase (0–5 min, panel **A**) or late phase (15–30 min, panel **B**) of formalin-induced nociception in mice. Each column represents the mean ± S.E.M. of six animals. Statistical differences between the treated and the control groups were evaluated by ANOVA and Dunnett’s test. The asterisks denote the level of significance in comparison with the control group, ** *p* < 0.01.

Neurogenic and inflammatory pain was evaluated using the formalin-induced nociception assay. In this test, BT-MeOH did not inhibit the neurogenic phase (Figure 3A), while the inflammatory phase was inhibited by 53.1% (66.8 ± 14.2 s; *p* < 0.01) (Figure 3B). Indomethacin inhibited the inflammatory phase by 60.2% (56.8 ± 8.7 s; *p* < 0.01). These data support the hot plate results, given that central analgesic drugs such as opioids are able to inhibit both phases in the formalin test, while NSAIDs and corticoids normally inhibit only the inflammatory phase [60,61]. The injection of formalin induces a biphasic behavioral response. The neurogenic phase begins shortly after the injection, occurring at about 5 min, and it results from the direct stimulation of nociceptors [62]. After a quiescent period, the inflammatory phase occurs between the 15th and 30th min. In this phase, there is release of inflammatory mediators (*i.e.*, histamine, prostaglandins, TNF-α and IL-1β) [63,64].

**Figure 4:**
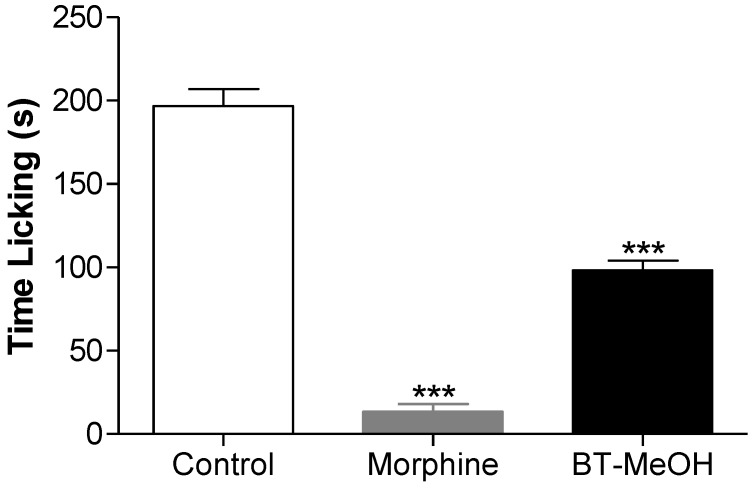
Antinociceptive effect of the BT-MeOH (100 mg/kg, p.o.) and morphine (5.7 mg/kg, s.c.) in glutamate-induced nociception test. Each column represents the mean ± S.E.M. of six animals. Statistical differences between the treated and control groups were evaluated by ANOVA and Dunnett’s test, and the asterisks denote the level of significance in comparison with control group, *** *p* < 0.001.

In addition, the algal extract was submitted to the glutamate-induced nociception test. BT-MeOH (100 mg/kg, p.o.) decreased the nociceptive response (50.1% inhibition) significantly (Figure 4). Glutamate is an excitatory neurotransmitter involved in nociceptive primary afferent transmission, and its action involves the activation of NMDA and non-NMDA receptors and depends on the activation of the L-arginine-nitric oxide pathway [65]. Besides its action in the development of the nociceptive response, both peripherally and centrally, its role in the maintenance of such process has been demonstrated by some studies [66,67,68]. This result indicates that BT-MeOH modulates the nociceptive action of the glutamatergic pathway.

**Figure 5:**
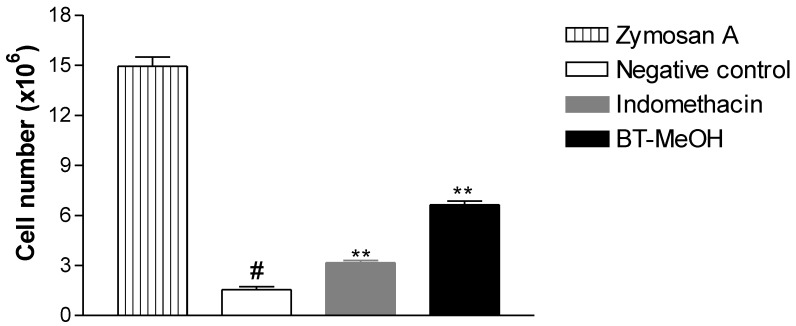
Anti-inflammatory effect of BT-MeOH (100 mg/kg, p.o) and indomethacin (35.7 mg/kg, p.o.) on zymosan A-induced peritonitis. Each point represents the mean ± S.E.M. of six animals. Statistical differences between the treated and control groups were evaluated by ANOVA and Dunnett’s test. The asterisks denote the level of significance in comparison with the zymosan group, ** *p* < 0.01, ^#^*p* < 0.01.

Zymosan A-induced peritonitis was used to evaluate the ability of BT-MeOH to inhibit leukocyte recruitment, one of the steps in the inflammatory process. The results obtained in this test showed that, in comparison with the zymosan A group (14.9 ± 0.5 × 10^6^ leukocytes/mL), BT-MeOH inhibited leukocyte migration by 55.6% (6.6 ± 0.2 × 10^6^ leukocytes/mL, *p* < 0.01), while indomethacin inhibited 78.1% (3.2 ± 0.1 × 10^6^ leukocytes/mL, *p* < 0.01) (Figure 5). This result corroborates the formalin results in which BT-MeOH was able to inhibit only the inflammatory phase.

Zymosan A is a cell wall polysaccharide of *Saccharomyces cerevisiae*, which is composed of β-glucans, mannans, mannoproteins and chitin. The zymosan A particles are recognized simultaneously by dectin-1, Toll-like receptor 2 and CD14. Once activated, these receptors trigger phagocytosis and production of reactive oxygen species (ROS) and induce signaling through NF-κB, which leads to the production of TNF-α, IL-8, IL12, and arachidonic acid products [69,70,71,72]. Taken together, these mediators trigger an inflammatory response induced by zymosan A. Furthermore, it is described in literature that H4 receptor antagonists are effective in reducing neutrophil influx in zymosan A-induced peritonitis [73,74,75]. Thus, there are some metabolites in BT-MeOH able to antagonize one or more steps of pathway signaling triggered by zymosan A.

Some studies have demonstrated an antioxidant action [76,77,78,79,80] and nitric oxide (NO) scavenging for *B. triquetrum* [79]. ROS, including molecules such as hydrogen peroxide, superoxide, hydroxyl radicals and NO, have been implicated in tissue damage and development of the inflammatory and pain response. Given that the phenolic compounds *trans*-cinnamic, *p*-coumaric and ferulic acids are the major constituents of *B. triquetrum* [80] and are antioxidant compounds capable of NO radical scavenging and attenuating the inflammatory and nociceptive process [81,82], the effect of BT-MeOH described in this study may be due, in part, to its antioxidant action. Furthermore, it was reported that sulfated carbohydrates from *Bryothamnion* species have antinociceptive activity [83,84]. Thus, these kinds of metabolites could contribute to the antinoceptive/anti-inflammatory action of BT-MeOH. Besides, the involvement of pigments such as phycocyanin in the antinociceptive/anti-inflammatory properties of *B. triquetrum* cannot be ruled out, since some studies have described such activities for this pigment [85,86].

## 3. Experimental Section

### 3.1. Plant Material

The alga *Bryothamnion triquetrum* (S. G. Gmelin) M.A. Howe was collected in the coastal region of Cabo Branco beach (7°08′52″S/34°48′13″W), João Pessoa, Paraíba State, Brazil in March 2010. The specimen was identified by Dr. George Emmanuel Cavalcanti de Miranda. Voucher specimens of *B. triquetrum*(JPB 47599) have been deposited in the Lauro Pires Xavier Herbarium at the Federal University of Paraiba, Brazil [87]. Fresh algae were lyophilized and exhaustively extracted with methanol in a Soxhlet apparatus, to obtain the respective extract, called BT-MeOH. 

### 3.2. Biological Activity Tests

#### 3.2.1. Drugs and Reagents

The following substances were used: acetic acid A.R. (Vetec); formaldehyde A.R (Vetec); zymosan A (Sigma); carboxymethylcellulose-CMC (Sigma); Tween^®^ 80 (Sigma-Aldrich); dipyrone (Sigma-Aldrich), indomethacin (Merck), morphine (Cristália, BR). A solution of 2.5% formalin was prepared with formaldehyde (Merck) in saline (0.9% NaCl). The extract was used as a suspension in Tween^®^ 80 (20 μL) and CMC (vehicle) in all experiments and were administered by the oral route (p.o.) at a dose of 100 mg/kg. Dipyrone, morphine and indomethacin were used as reference drugs. 

#### 3.2.2. Animals

Swiss mice of both sexes, 6–8 weeks of age with an average weight 25–30 g, were obtained from the Central Animal House of the Federal University of Alagoas (Maceió, Brazil) and were used throughout the experiments. They were housed in single-sex cages under a 12-h light/dark cycle at constant temperature (22 ± 2 °C) conditions with free access to water and pellet food. Eight hours before each experiment, the animals received only water, to avoid food interference with test substance absorption. The experiments were performed after the approval of the protocol by the Ethics Committee-UFAL for animal handling (No. 23065.017275/2011-94). 

#### 3.2.3. Acute Toxicity Study

This assay was conducted as described by Almeida *et al.* [88] with minor modifications. Two groups of six Swiss mice (six males and six females) after fasting for 12 h were treated with BT-MeOH, p.o., the route of administration used in all experiments. The acute toxicity of BT-MeOH was determined using the following doses: 100, 500 and 1000 mg/kg, starting with the one most likely to cause death (1000 mg/kg). If more than one animal died, the doses were administered sequentially decreasing the power estimate to a range of LD_50_ (50% lethal dose). During the first 24 h, for periods of 0, 15, 30 and 60 min every 4 h, and daily for 14 days after the administration of BT-MeOH, the following behavioral parameters were determined: changes in locomotion, drowsiness, piloerection, diarrhea, salivation, writhings, convulsions, hyperexcitability and death of animals. At the end of the observation period, all survivors were sacrificed and autopsied.

#### 3.2.4. Acetic Acid-Induced Writhing

The test was carried out using the method previously described by Collier *et al.* [89]. Abdominal writhing in mice was induced by intraperitoneal (i.p.) injection of acetic acid (0.6% solution, 0.1 mL/10 g). The animals were pre-treated with BT-MeOH (100 mg/kg, p.o.), CMC/Tween^®^ 80 (vehicle) or dipyrone (33.3 mg/kg, p.o.) 40 min before initiating algesic stimulation (*n* = 6/group). The writhing response, which consists of a contraction of the abdominal muscle together with a stretching of the hind limbs, was determined for 20 min after a latency period of 5 min. 

#### 3.2.5. Hot-Plate Test

The test was performed as described by Kuraish *et al.* [58]. Different groups of animals (*n* = 6/group) received BT-MeOH (100 mg/kg, p.o.), vehicle or morphine (5.7 mg/kg, s.c.). After 40 min, the mice were placed on a hot plate maintained at 54 ± 1 °C, separated by a 30-min interval. The baseline was considered as the reaction time obtained at 30 min before the administration and was defined as the normal reaction of the animal to temperature. After treatment, the reaction time when the animals licked their fore and hind paws and jumped was recorded at 30, 60, 90 and 120 min. The cut-off time used to prevent skin damage was 15 s. 

#### 3.2.6. Formalin-Induced Nociception

The procedure described by Hunskaar and Hole [62] was followed with slight modifications. Mice were pre-treated with BT-MeOH (100 mg/kg, p.o.), indomethacin (35.7 mg/kg, p.o.) or vehicle, 40 min before intraplantar injection of 2.5% formalin solution (20 μL) into the right hind paw of the animal (*n* = 6/group). The response was the time which the animals spent licking the injected paw. Two distinct periods of high licking activity can be identified, a neurogenic phase during the first 5 min and an inflammatory phase lasting between 15 and 30 min after the injection of formalin.

#### 3.2.7. Glutamate-Induced Nociception

A volume of 20 μL of glutamate solution (30 μmol/paw prepared in saline) was intraplantarly injected in the ventral surface of the right hindpaw. Animals were observed individually for 15 min after glutamate injection. The time that they spent licking the injected paw was recorded and was considered as indicative of nociception. Animals were treated with BT-MeOH (100 mg/kg, p.o.), morphine (5.7 mg/kg, s.c.) or vehicle 40 min before glutamate injection [65].

#### 3.2.8. Zymosan A-Induced Peritonitis

This test was conducted as described by Doherty *et al.* [90]. Leukocyte migration was induced by injection of zymosan A (1 mg/animal, i.p.) 40 min after administration of BT-MeOH (100 mg/kg, p.o., *n* = 6), indomethacin (35.7 mg/kg, p.o., *n* = 6) or vehicle (*n* = 6). Six hours after zymosan A injection, the animals were euthanized by cervical dislocation. Shortly afterwards, the peritoneal cavity was washed with cold PBS (3.0 mL, i.p.), and after gentle manual massage, the exudate was withdrawn (around 2.0 mL). The total cells were counted in a Neubauer chamber, under light microscopy at 40×. The results were expressed as the number of leukocytes/mL.

#### 3.2.9. Statistical Analysis

Data obtained from animal experiments were expressed as mean and standard error of the mean (mean ± S.E.M.). Statistical differences between the treated and control groups were evaluated by ANOVA followed by Dunnett’s test. A value of *p* < 0.05 was considered statistically significant.

## 4. Conclusions

Taken together, the results obtained with our tests suggest that BT-MeOH contains metabolites able to modulate the peripheral nociceptive and inflammatory processes. However, more studies must be conducted to confirm such properties.
